# Supplementary material for: Detailed measurements and simulations of electric field distribution of two TMS coils cleared for obsessive compulsive disorder in the brain and in specific regions associated with OCD
Source: PLoS One. 2022 Aug 30;17(8):e0263145. doi: 10.1371/journal.pone.0263145 (PMC9426893; doi:10.1371/journal.pone.0263145)
Supplement: S1 Table — (DOCX) [file pone.0263145.s001.docx]

**S1 Table: Electrical conductivity (S/m) values used for the PHM models’ simulations.**

| **Tissue** | **PHM v.1** | **PHM v.2** |
| --- | --- | --- |
| WM | 0.265 | 0.126 |
| GM | 0.239 | 0.275 |
| Cerebellum | 0.659 | 0.126 |
| CSF | 1.777 | 1.654 |
| Skull | 0.082 | 0.010 |
| Skin | 0.170 | 0.465 |
