# Supplementary material for: Detailed measurements and simulations of electric field distribution of two TMS coils cleared for obsessive compulsive disorder in the brain and in specific regions associated with OCD
Source: PLoS One. 2022 Aug 30;17(8):e0263145. doi: 10.1371/journal.pone.0263145 (PMC9426893; doi:10.1371/journal.pone.0263145)
Supplement: S2 Table — Results of comparison of the electric field decay profile with distance between the D-B80 and the H7 coils using Wilcoxon matched-paired test, for simulations in 22 head models. (DOCX) [file pone.0263145.s002.docx]

**S2 Table: Comparison of electric field decay profiles.** Results of comparison of the electric field decay profile with distance between the D-B80 and the H7 coils using Wilcoxon matched-paired test, for simulations in 22 head models.

| Model | | p |
| --- | --- | --- |
| Homogeneous | Thelonious | 2.13E-16 |
|  | Ella | 2.56E-11 |
|  | Duke | 3.30E-04 |
| ViP | Thelonious | 2.18E-17 |
|  | Ella | 1.88E-08 |
|  | Duke | 8.14E-03 |
| PHM v.1 | #101309 | 1.36E-07 |
|  | #103111 | 5.14E-11 |
|  | #103414 | 7.62E-09 |
|  | #105014 | 1.05E-07 |
|  | #105115 | 1.09E-06 |
|  | #106016 | 1.11E-11 |
|  | #110411 | 1.92E-06 |
|  | #111716 | 2.23E-03 |
| PHM v.2 | #101309 | 3.15E-08 |
|  | #103111 | 5.14E-11 |
|  | #103414 | 7.62E-09 |
|  | #105014 | 1.01E-08 |
|  | #105115 | 9.63E-09 |
|  | #106016 | 1.11E-11 |
|  | #110411 | 7.74E-07 |
|  | #111716 | 5.76E-04 |
