# Supplementary figures and images for: Detailed measurements and simulations of electric field distribution of two TMS coils cleared for obsessive compulsive disorder in the brain and in specific regions associated with OCD
Source: PLoS One. 2022 Aug 30;17(8):e0263145. doi: 10.1371/journal.pone.0263145 (PMC9426893; doi:10.1371/journal.pone.0263145)

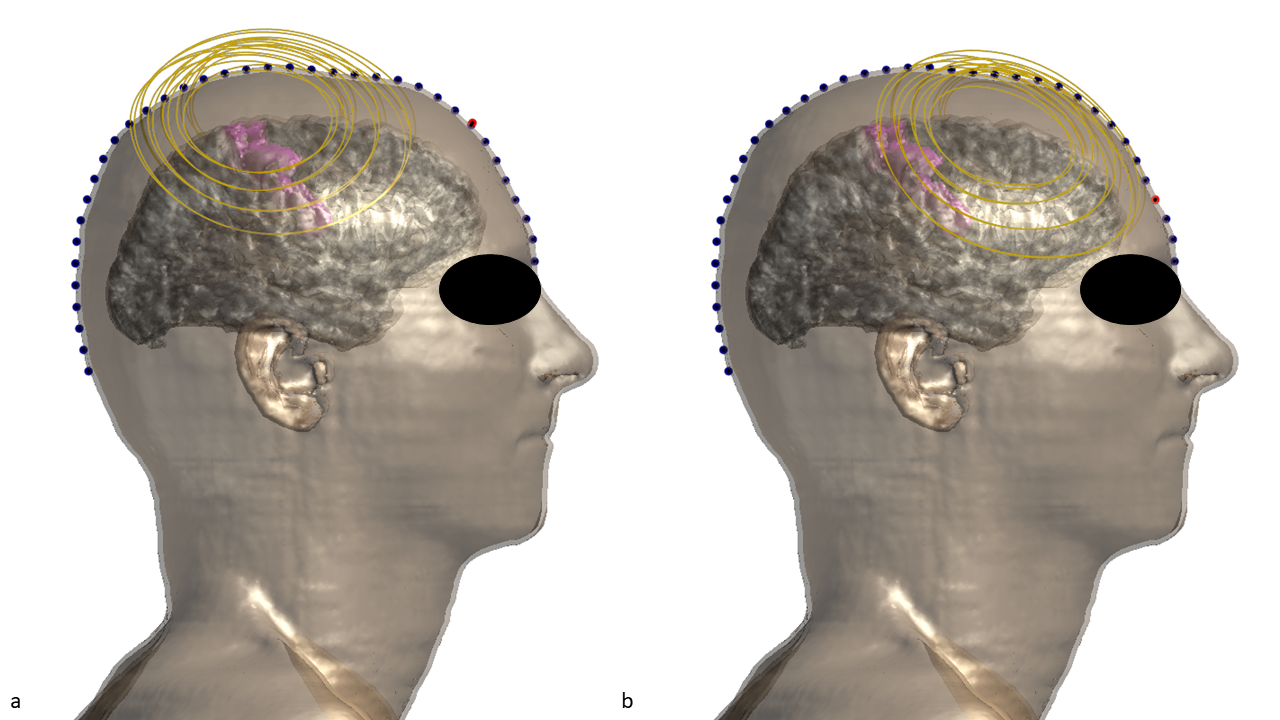

Supplement: S1 Fig — Coil placement process over the head model of Duke. The reference point of the H7 coil, indicated with a red dot, is placed 8 cm from the nasion, along the nasion-inion measuring spline, as in clinical practice (a). There the MC is visually identified as BA 4 and the coil position is manually adjusted, if needed. Then, the coil is moved 4 cm anteriorly, over the PFC (b). (TIF) [file pone.0263145.s003.tif]

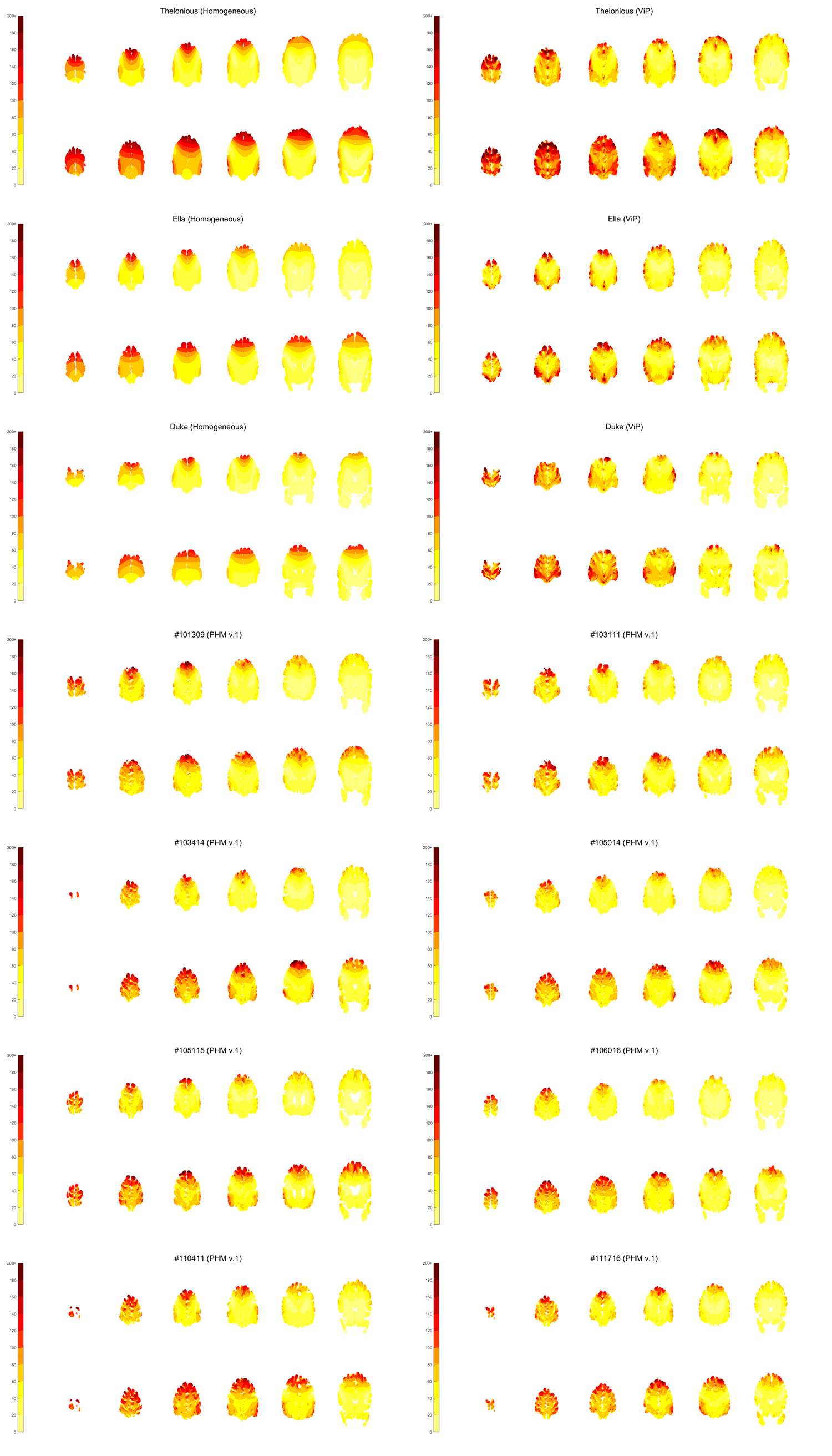

Supplement: S2 Fig — Colored field maps for the D-B80 coil (top row) and for the H7 coil (bottom row) showing electric field distribution within the brain, when located at the treatment location over the prefrontal cortex, indicating the electrical field absolute magnitude in each pixel over the 6 most frontal coronal slices 1 cm apart. The red pixels indicate field magnitude ≥ the threshold for neuronal activation, which was set to 100 V/m. Shown are results for the 14 numerical head models. (TIF) [file pone.0263145.s004.tif]

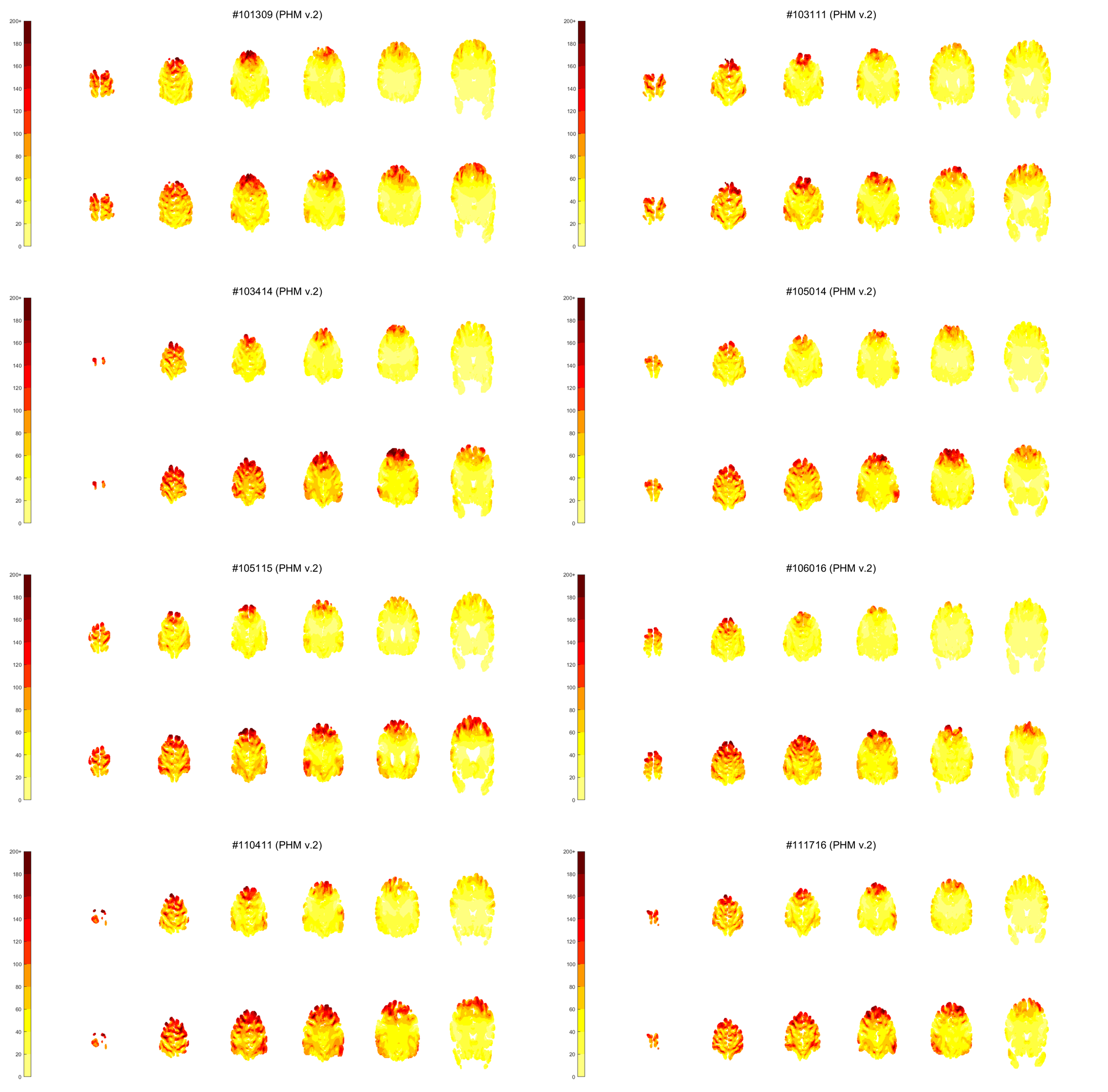

Supplement: S3 Fig — Colored field maps for the D-B80 coil (top row) and for the H7 coil (bottom row) showing electric field distribution within the brain, when located at the treatment location over the prefrontal cortex, indicating the electrical field absolute magnitude in each pixel over the 6 most frontal coronal slices 1 cm apart. The red pixels indicate field magnitude ≥ the threshold for neuronal activation, which was set to 100 V/m. Shown are results for the 8 PHM v.2 simulated head models. (TIF) [file pone.0263145.s005.tif]

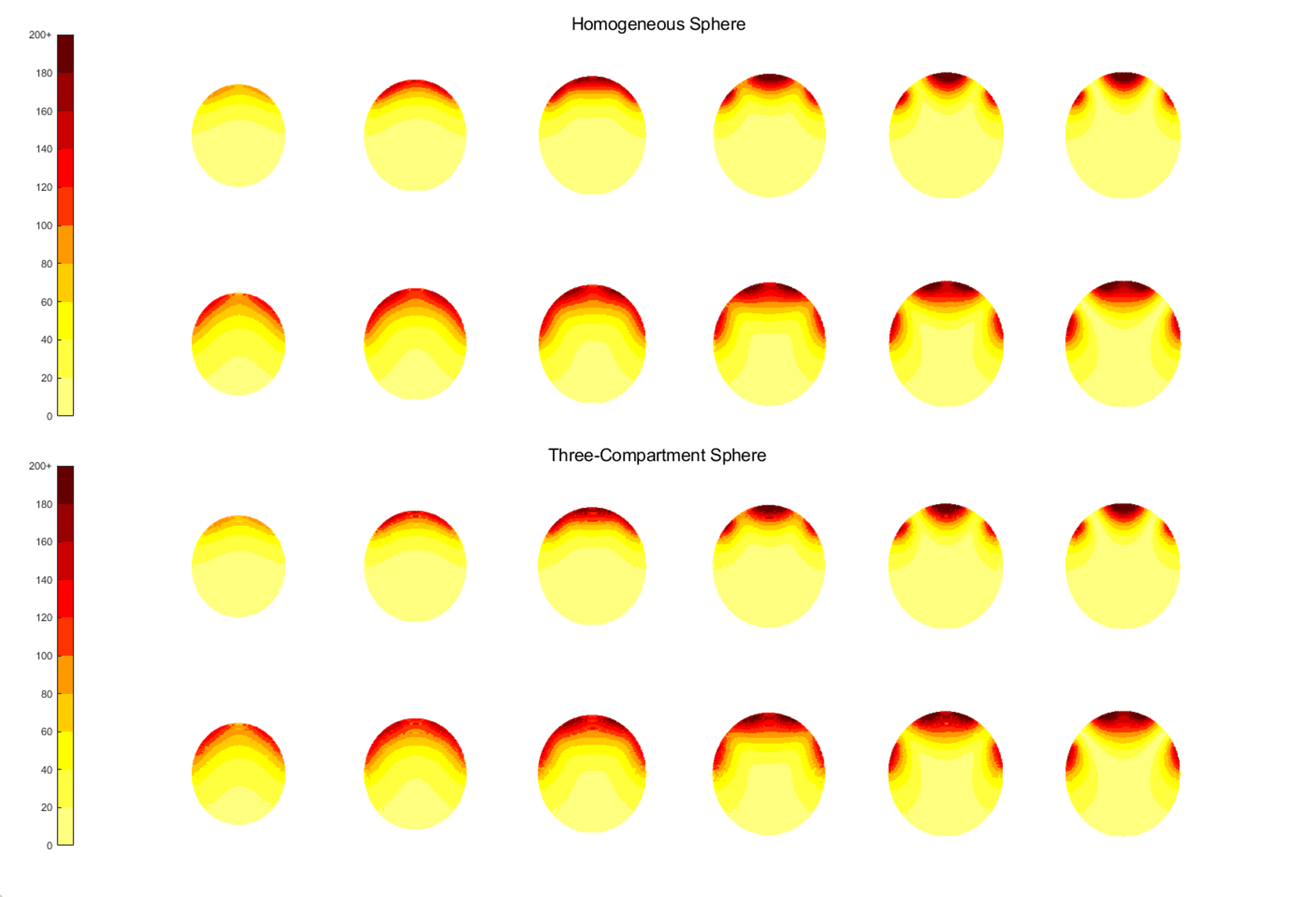

Supplement: S4 Fig — Colored field maps for the D-B80 coil (top row) and for the H7 coil (bottom row) showing electric field distribution within the brain, when located at the treatment location over the prefrontal cortex, indicating the electrical field absolute magnitude in each pixel over the 6 most frontal coronal slices 1 cm apart. The red pixels indicate field magnitude ≥ the threshold for neuronal activation, which was set to 100 V/m. Shown are results for the 2 spherical models. (TIF) [file pone.0263145.s006.tif]

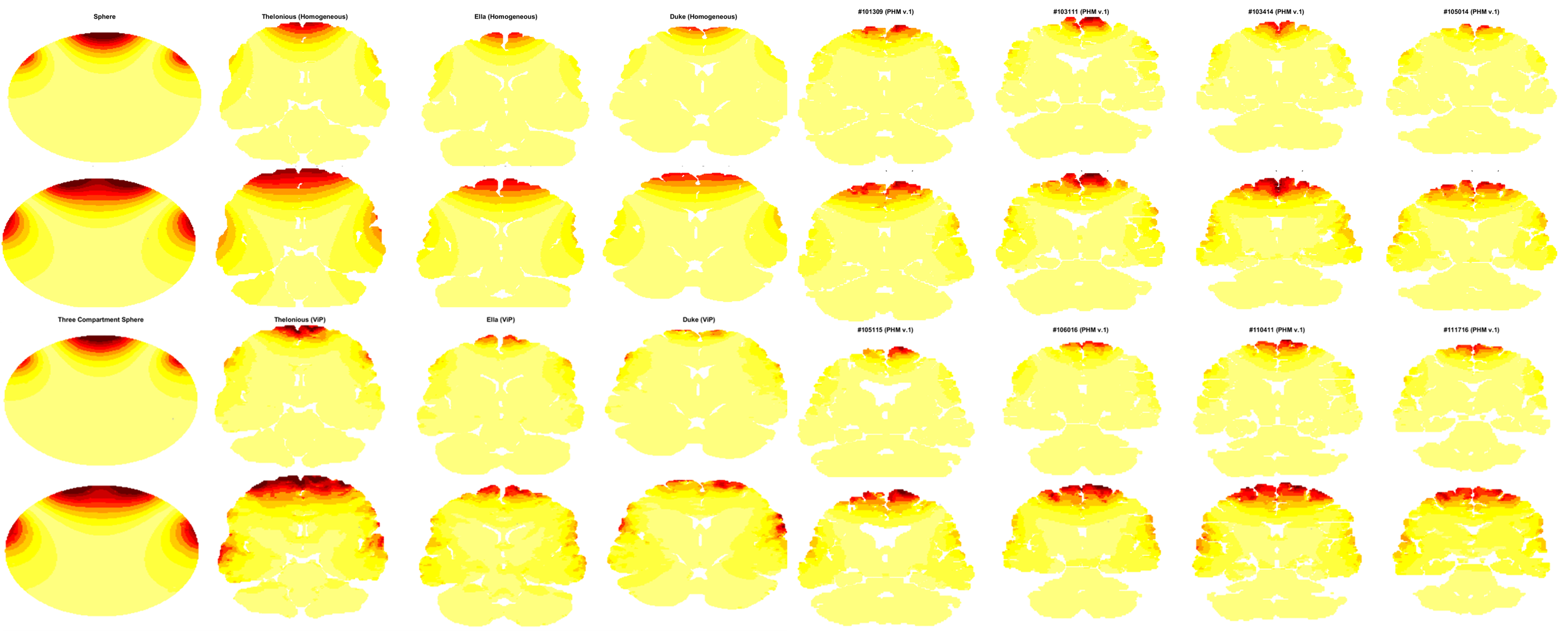

Supplement: S5 Fig — Colored field maps for the D-B80 coil (top row) and for the H7 coil (bottom row) showing electric field distribution on a slice perpendicular to the coil center, when located at the treatment location over the prefrontal cortex, indicating the electrical field absolute magnitude in each pixel. The red pixels indicate field magnitude ≥ the threshold for neuronal activation, which was set to 100 V/m. Shown are results for the 14 numerical head models and the two spherical models. (TIF) [file pone.0263145.s007.tif]

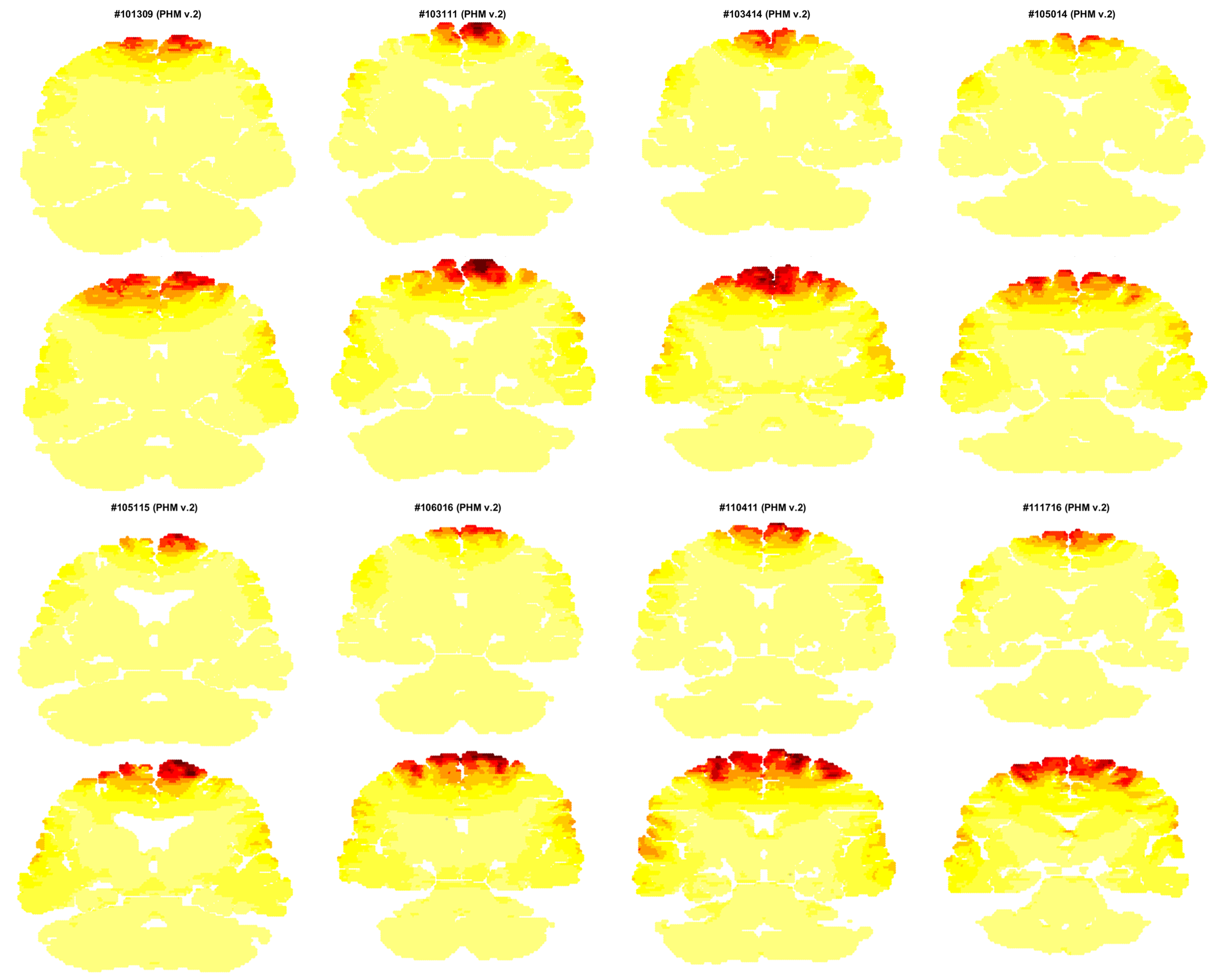

Supplement: S6 Fig — Colored field maps for the D-B80 coil (top row) and for the H7 coil (bottom row) showing electric field distribution on a slice perpendicular to the coil center, when located at the treatment location over the prefrontal cortex, indicating the electrical field absolute magnitude in each pixel. The red pixels indicate field magnitude ≥ the threshold for neuronal activation, which was set to 100 V/m. Shown are results for the 8 PHM v.2 simulated head models. (TIF) [file pone.0263145.s008.tif]

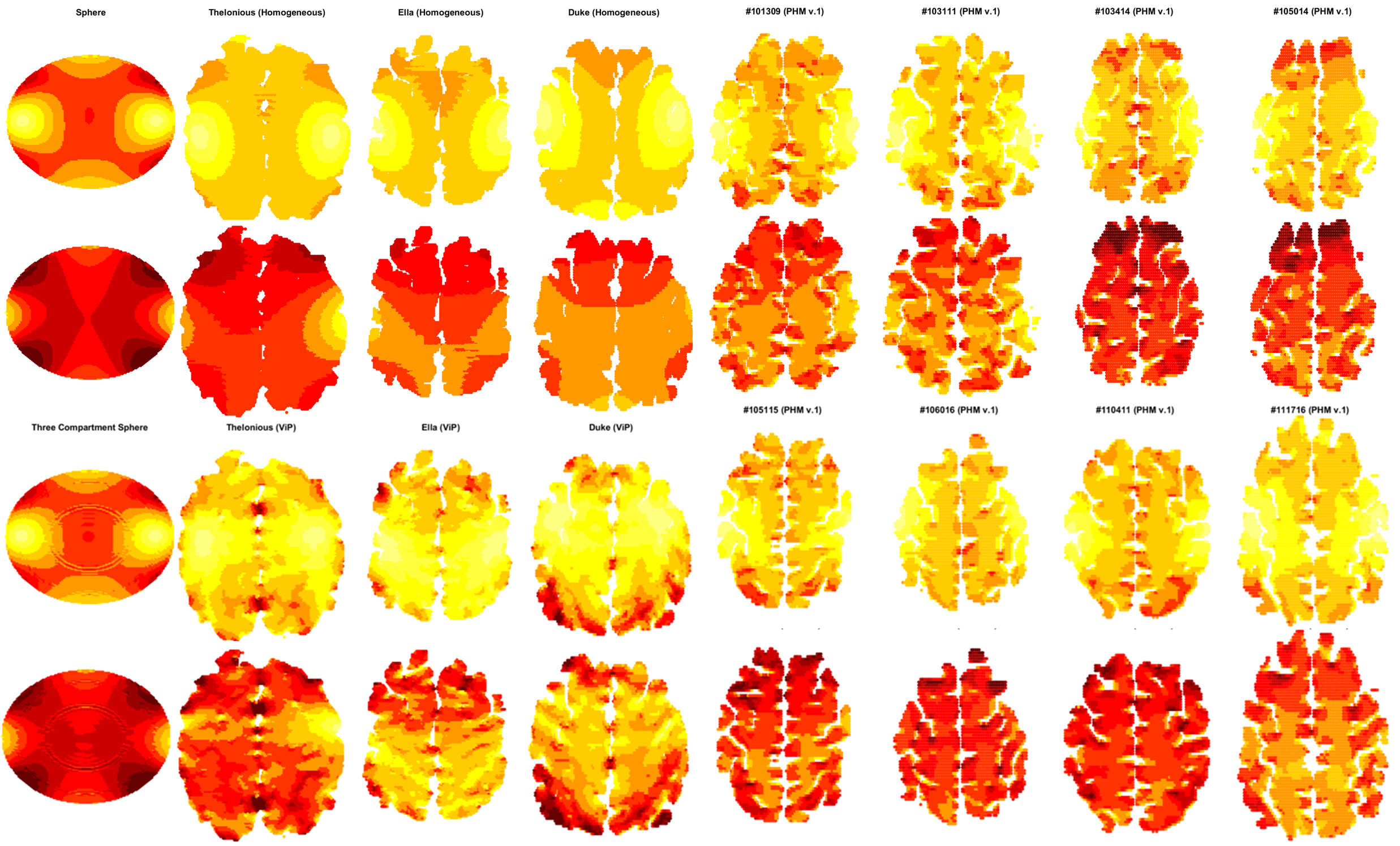

Supplement: S7 Fig — Colored field maps for the D-B80 coil (top row) and for the H7 coil (bottom row) showing electric field distribution on a slice parallel to the coil at a 2 cm distance, when located at the treatment location over the prefrontal cortex, indicating the electrical field absolute magnitude in each pixel. The red pixels indicate field magnitude ≥ the threshold for neuronal activation, which was set to 100 V/m. Shown are results for the 14 numerical head models and the two spherical models. (TIF) [file pone.0263145.s009.tif]

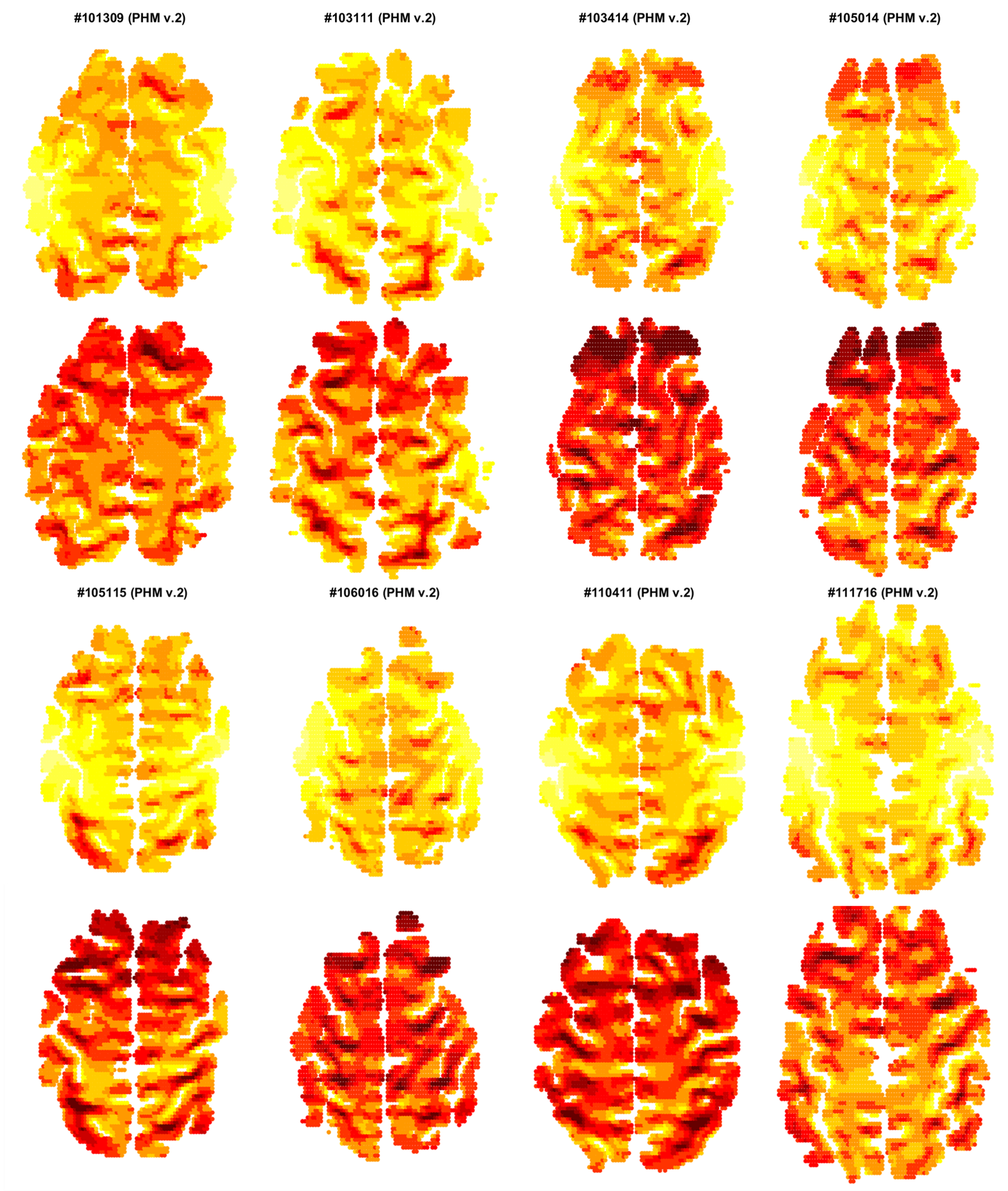

Supplement: S8 Fig — Colored field maps for the D-B80 coil (top row) and for the H7 coil (bottom row) showing electric field distribution on a slice parallel to the coil at a 2 cm distance, when located at the treatment location over the prefrontal cortex, indicating the electrical field absolute magnitude in each pixel. The red pixels indicate field magnitude ≥ the threshold for neuronal activation, which was set to 100 V/m. Shown are results for the 8 PHM v.2 simulated head models. (TIF) [file pone.0263145.s010.tif]

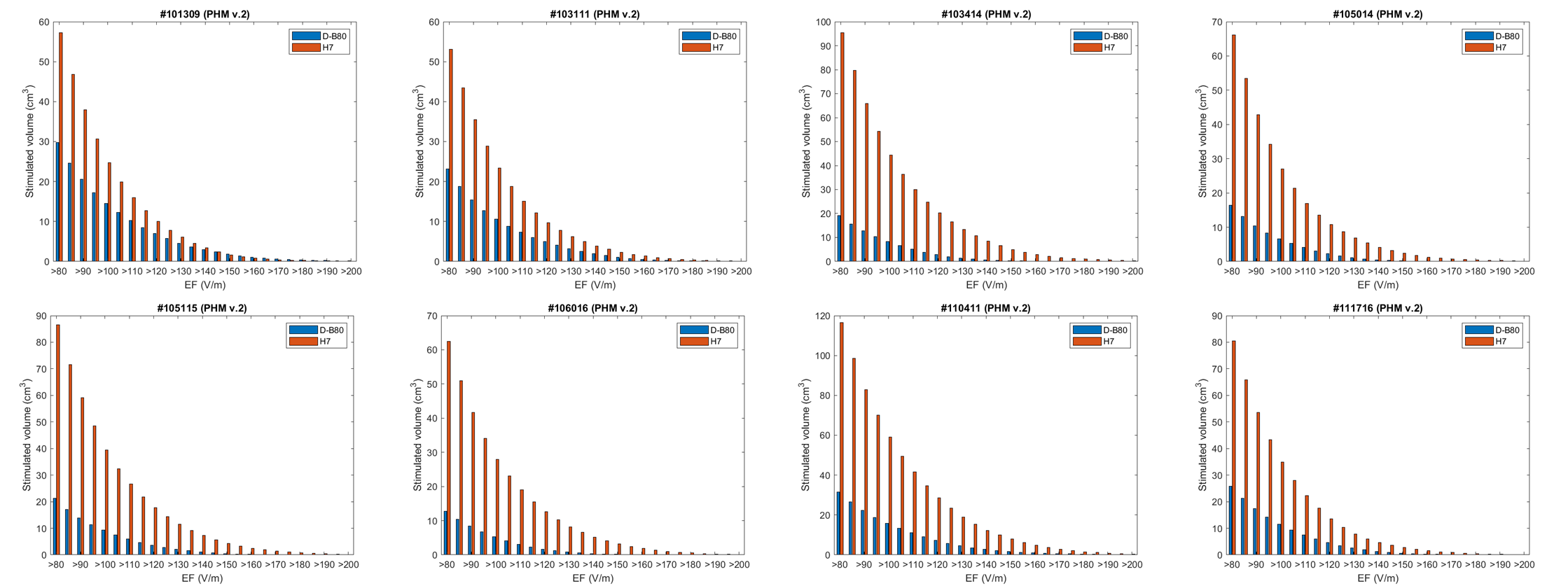

Supplement: S9 Fig — Histograms of distribution of the number of pixels within the brain according to the induced electric field range for the D-B80 and the H7 coil. Field columns are in bins of 5 V/m. Shown results for the 8 PHM v.2 simulated head models. (TIF) [file pone.0263145.s011.tif]

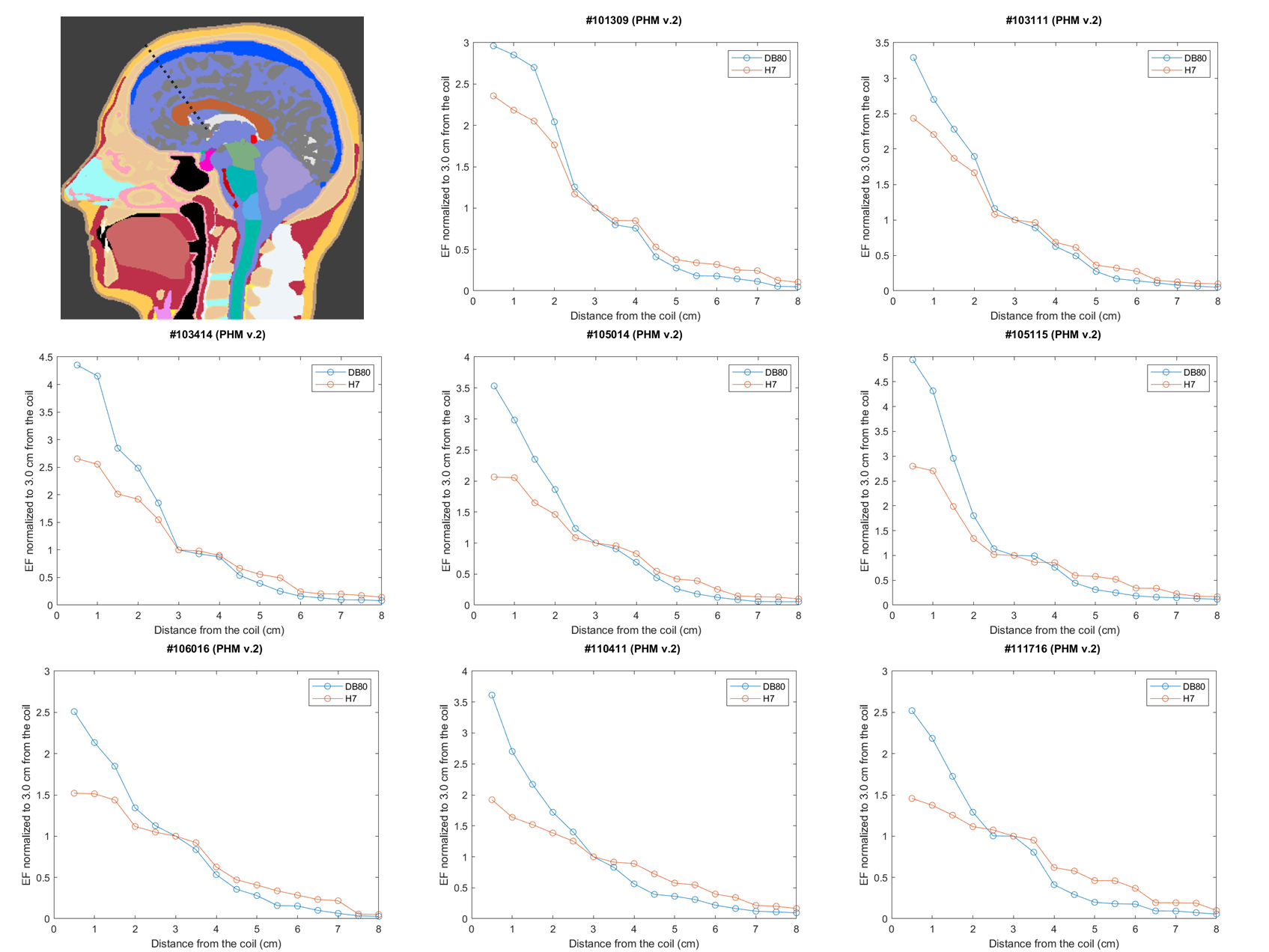

Supplement: S10 Fig — The electric field magnitude as a function of the distance from the coil, is shown for the D-B80 coil and for the H7 coil, when located at the treatment location over the prefrontal cortex. The field was measured along a line in a central sagittal plane starting at the point of inflection of the frontal bone and going at 45° downward and posteriorly (top left). The electric field was normalized to the field at a depth of 3 cm from coil surface. Shown results for the 8 PHM v.2 simulated head models. (TIF) [file pone.0263145.s012.tif]
